# LARS2 Regulates Apoptosis via ROS-Mediated Mitochondrial Dysfunction and Endoplasmic Reticulum Stress in Ovarian Granulosa Cells

**DOI:** 10.1155/2022/5501346

**Published:** 2022-05-09

**Authors:** Shujun Feng, Shan Wan, Shuangying Liu, Wei Wang, Minyue Tang, Long Bai, Yimin Zhu

**Affiliations:** ^1^Department of Reproductive Endocrinology, Women's Hospital, School of Medicine, Zhejiang University, Hangzhou, Zhejiang 310006, China; ^2^Key Laboratory of Reproductive Genetics (Ministry of Education) and Women's Reproductive Health Laboratory of Zhejiang Province, Women's Hospital, School of Medicine, Zhejiang University, Hangzhou, Zhejiang 310006, China

## Abstract

Several studies have indicated that mutations of LARS2 are associated with premature ovarian insufficiency (POI). However, the pathogenic mechanism of LARS2 in POI has not been reported yet. In the present study, the expression levels of LARS2 and E2F1 in granulosa cells (GCs) of POI patients were examined. CCK-8 and Edu assay were performed to determine the effect of LARS2 on cell proliferation. Apoptosis rate, mitochondrial membrane potential, reactive oxygen species (ROS), and cytoplasm Ca^2+^ levels were analyzed by flow cytometry. Western blot was conducted to evaluate the expression level of genes affected by LARS2. Transmission electron microscopy (TEM) was used to observe mitochondrial structure in GCs. Chromatin immunoprecipitation (ChIP) was used to evaluate the regulatory effect of E2F1 on Mfn-2 expression. Our results showed that LARS2 expression was downregulated in GCs of POI patients. Silencing of LARS2 inhibited cell proliferation and promoted the apoptosis of GCs. Meanwhile, LARS2 knockdown could induce mitochondrial dysfunction and accumulation of ROS levels. Moreover, ROS was found to be involved in the antiproliferation, proapoptotic, and endoplasmic reticulum (ER) stress effects of LARS2 knockdown. Furthermore, we also found that the expression level of E2F1 was positively correlated with LARS2. In addition, E2F1 could bind at the -61/-46 region of Mfn-2 promoter and regulated MFN-2 transcription. These findings demonstrated that LARS2 could promote the expression of E2F1. E2F1 mediated the effect of LARS2 on Mfn-2 expression via targeting the promoter region of Mfn-2, in which subsequently regulated cell proliferation and apoptosis, which resulted in the etiology of POI. This study will provide useful information for further investigations on the LARS2 in the occurrence of POI.

## 1. Introduction

Primary ovarian insufficiency (POI) is a reproductive endocrine disease exhibiting severely impaired ovary function with amenorrhea before the age of 40 [1].

Multiple factors have been identified as etiologies of POI, including genetics, environment, and chemotherapeutic treatment. However, the pathogenesis of POI is still difficult to identify. In clinic, POI patients showed high follicle-stimulating hormone (FSH) levels (FSH>25 mIU/mL on two occasions>4 weeks apart) and low estradiol (E2) levels [2]. In follicle development phase, cell-cell crosstalk between oocyte and surrounding somatic cell is crucial for maintaining proper hormone levels. Granulosa cell is the main cell type in follicle communicating with oocyte directly through jap junctions or in a paracrine manner. Particularly, granulosa cell could be responding for FSH by expressing FSH receptor and producing E2 to guarantee normal menstrual cycle. It has been reported that impaired granulosa cell function contributes to the occurrence of POI [3]. Thus, the exploration of mechanism involved in granulosa cell function disruption will be a benefit for the diagnosis of POI.

The mitochondrion is the core organelle of a cell that is well known for its function in generating ATP through the oxidative phosphorylation process. Besides working as the powerhouse of a cell, mitochondria participate in various critical cellular functions, including lipid synthesis, calcium handling, production of ROS, and control of cell death [4]. Accordingly, the disrupted mitochondrial function will induce the pathology of a follicular somatic cell (particularly granulosa cell), which may lead to the occurrence of POI. In fact, mitochondrial dysfunction-linked POI has been reported. Thus far, several genes have been demonstrated that are associated with POI by regulating mitochondria function [5]. Leucyl-tRNA synthetase 2 (LARS2) is an aminoacyl-tRNA synthetase controlling the translation of mitochondrial-encoded genes through encoding the precursor of mitochondrial leucyl-tRNA synthetase. 1 LARS2 is an essential mediator, that regulating of process of charging of tRNA Leu (UUR) with leucine. [6]. Meanwhile, LARS2 is also indirectly required for mitochondrial genome maintenance, in which LARS2 regulates group I intron RNA splicing and protein synthesis of mitochondrion [7]. The aberrant expression of LARS2 in cancer cells has been demonstrated [8]. LARS2 is involved in the regulation of cell apoptosis [9]. Furthermore, it has been reported that the mutations in LARS2 gene are associated with the occurrence of Perrault syndrome, which describes a rare recessively inherited condition composed of hearing loss and ovarian dysfunction in females including ovarian dysgenesis and primary amenorrhea [10, 11]. However, the underlying mechanism of LARS2-induced POI pathology remains unknown. As the important component of the follicle, normal granulosa cell physiology is responsible for the maintenance of the folliculogenesis process. The apoptosis of granulosa cell will cause the occurrence of POI. To date, few studies have reported the role of LARS2 in granulosa cell biology. The role of LARS2 in cancer indicates that aberrant expression of LARS2 might cause the occurrence of POI via inducing granulosa cell apoptosis. In the present study, the differential expression of LARS2 in granulosa cells between POI and control groups were explored. Meanwhile, the effect of LARS2 on granulosa cell proliferation, apoptosis, mitochondrial function, and underlying mechanism was also investigated.

## 2. Materials and Methods

### 2.1. Ethics Statement and Human Subjects

The study was approved by the Ethical Committee of the Women's Hospital, Zhejiang University School of Medicine, China (file no. 20180139). All participants signed a document of informed consent before participating in the study. All subjects were obtained from women undergoing IVF-embryo transfer (IVF-ET) at the Center for Reproductive Medicine, Women's Hospital, Zhejiang University School of Medicine. The clinical characteristics of all the participants are shown in Table 1. Inclusion criteria for the patients in the POI group were included: (a) AMH<1.1 ng/mL, (b) over 40 years old, and (c) the number of obtained oocyte<3, according to any two of the above standards. The women recruited into the control group were included: (a) regular menstrual cycle, (b) basal FSH (on days 2-4 of the menstrual cycle) <10 IU/L, and (c) the patient sought IVF treatment due to male factors or tubal obstruction.

### 2.2. Animals Study

All animal studies were approved by the Zhejiang University Animal Care and Use Committee (File no. ZJU20210055). The 3-week C57BL/6 female mice were provided by the laboratory animal center of Zhejiang University and randomly divided into three groups, named control, POI, and treatment groups. Each group contains five mice. POI and treatment groups were given an intraperitoneal injection of doxorubicin (10 mg/kg) once, whereas the control group received an intraperitoneal injection of 0.9% physiological saline. After 7 days of injection, the treatment group was injected with GH (1.6 mg/kg) for additional 21 days. Meanwhile, the POI group was treated with 0.9% physiological saline. Then all the mice were sacrificed, and ovary tissues were collected. One side of the ovary was fixed in 4% paraformaldehyde until H&E staining. On the other side of the ovary was extracted protein.

### 2.3. Cell Lines and Cell Culture

KGN were purchased from the cell bank of the Chinese Academy of Sciences (Shanghai, China). The cells were cultured in DMEM (HyClone, USA) supplemented with 10% fetal bovine serum (BI, USA) and 100 IU/mL penicillin and streptomycin (Gibco, USA) at 37°C with 5% CO_2_. All cells were tested negative for mycoplasma contamination.

The 3-week C57BL/6 female mice were injected with 10 units of PMSG and sacrificed 44 h later. Ovaries were obtained from superovulated mice and transferred into Petri dishes filled with PBS and then pricked with a syringe under a surgical dissecting microscope to release granulosa cells. The cell suspensions were put into cell culture plate containing DMEM/F-12 with 10% fetal bovine serum and incubated at 37°C with 5% CO_2_.

### 2.4. Cell Transfection

LARS2 siRNAs (si-LARS2-1; si-LARS2-2) and one scrambled control siRNA were purchased from GenePharma (Shanghai, China). Cells were seeded into 6-well plates or 12-well plates and transfected with si-LARS2, scrambled siRNAs using Lipofectamine iMAX (Invitrogen, USA). The siRNA sequences were shown as following: si-LARS2-1, sense: 5′-GGACUUCACAUUAAAGGUUTT-3′; antisense: 5′-AACCUUUAAUGUGAAGUCCTT-3′; si-LARS2-2, sense: 5′-CCACGAAUUUG UUCUUCAATT-3′; antisense: 5′-UUGAAGAACAAAUUCGUGGTT-3′. After 48-h transfection, the cells were collected for mRNA or protein analysis.

### 2.5. RNA Extraction, Reverse Transcription, and Quantitative Real-Time PCR

Total RNA was extracted from cells, and tissue was exacted with TRIzol reagents (Takara, Japan). RNA was reverse transcribed into the complementary DNA (cDNA) using PrimeScript RT reagent Kit (Takara, Japan) according to the instruction. Each 10 *μ*L sample volume containing 5.4 *μ*L SYBR Green PCR Master Mix (Takara, Japan), 0.2*μ*l each specific primer, and 25 ng of cDNA was determined by quantitative real-time PCR (qRT-PCR). The specific primers used were shown in Table 2. GAPDH was used as a reference gene. The relative expression levels of target gene mRNA were displayed using the comparative Ct method with the 2^−*ΔΔ*Ct^ values formula. The specific primer sequences are listed in Table 2. All primers were designed by Sangon Biotech Online Primer Design Tool (Shanghai, China).

### 2.6. Western Blot

Lysis of cells and tissues were collected by RIPA buffer (Solarbio, China). The isolation and extraction of cytoplasmic protein was using a Cell Mitochondria Isolation Kit (Beyotime, China) according to the manufacturer's instructions. The protein concentration was measured with a BCA protein assay kit (Thermo Fisher, USA). Equal amounts of protein were separated in sodium dodecyl sulfate-polyacrylamide gel electrophoresis (SDS-PAGE) and transferred onto polyvinylidene fluoride (PVDF) membranes. The membrane was blocked with 5% skim milk for 2 h and incubated with the primary antibody. The antibodies were obtained from different companies including anti-LARS2 (17097-1-AP), anti-Bax (50599-2-Ig), anti-Bcl-2 (12789-1-AP), anti-cytochrome C (10993-1-AP), anti-GRP78 (11587-1-AP), and anti-CHOP (15204-1-AP) from Proteintech and anti-Cleaved Caspase-3 (14220 T), anti-phospho-ERK1/2 (4370 T), anti-ERK1/2 (4695 T), anti-phospho-AKT (4060 T), and anti-AKT (4691 T) from Cell Signaling Technology. The next day, membranes were incubated in the appropriate HRP-conjugated secondary antibody for 1 h. After the removal of excess antibodies by washing, specific binding was detected using an ECL kit (Affinity, USA). An anti-*β*-actin antibody (ZSGQ-Bio, China) was used as a loading control.

### 2.7. ROS Detection

ROS was detected by means of the ROS detection assay kit (Beyotime, China) according to the manufacturer's instructions. Cells were stained in 1 : 1000 DCFH in PBS for 20 min at room temperature. The mitochondrial ROS level was detected by using MitoSOX (Invitrogen, USA). Five *μ*M MitoSOX reagent working solution was added to the cells. The cells were incubated for 20 min at 37°C. Then, the cells were analyzed using the CytExpert software in the flow cytometer CytoFLEX (Beckman, USA).

### 2.8. Cell Apoptosis Analysis

Cells were collected by trypsinization (without EDTA). Then, the cells were stained with fluorescein isothiocyanate (FITC)-Annexin V and propidium iodide (PI) by using the FITC Annexin V and PI apoptosis detection kit (Solarbio, China). The stained cells were analyzed by flow cytometer CytoFLEX (Beckman, USA). Next, the relative number of Annexin V- or PI-positive cells was calculated and compared.

### 2.9. ATP Quantification

ATP was determined with the ATP Testing Assay Kit (Beyotime, China) according to the manufacturer's instructions. Cells in 12-well plates were lysed in ATP lysis buffer and centrifuged at 12,000 ×*g* for 10 min. Supernatants were mixed with a testing buffer, and ATP concentrations were measured on a luminescence detector.

### 2.10. Hematoxylin and Eosin Stain

Ovaries were fixed in 4% paraformaldehyde in Dulbecco's phosphate-buffered saline (DPBS; Sigma) at room temperature overnight and stored at 4°C in fresh 70% ethanol until processed. Ovaries were then dehydrated and embedded in paraffin, and 5-*μ*m serial sections were stained with H&E using standard protocol. All sections were observed under an optical microscope (Nikon, Japan).

### 2.11. Chromatin Immunoprecipitation (ChIP) Assays

ChIP assays were performed with Simple ChIP Plus Enzymatic ChIP Kit from Cell Signaling Technology (9005S, USA) according to the manufacturer's instructions. Quantitative PCR (qPCR) analysis was performed to detect the DNA fragments that coimmunoprecipitated with E2F1. The specific primer used for the E2F1 binding site within the Mfn-2 promoter was shown in Table 2. The primers were designed by Sangon Biotech Online Primer Design Tool (Shanghai, China).

### 2.12. Statistical Analysis

All experiments were performed with at least three independent replicates. Data are presented as the mean ± SD of three independent experiments. Group comparisons were conducted by two-tailed unpaired Student's *t*-tests. *P* value of <0.05 was considered significant.

## 3. Results

### 3.1. LARS2 Expression Levels Are Lower in POI Patients

**Figure 1:**
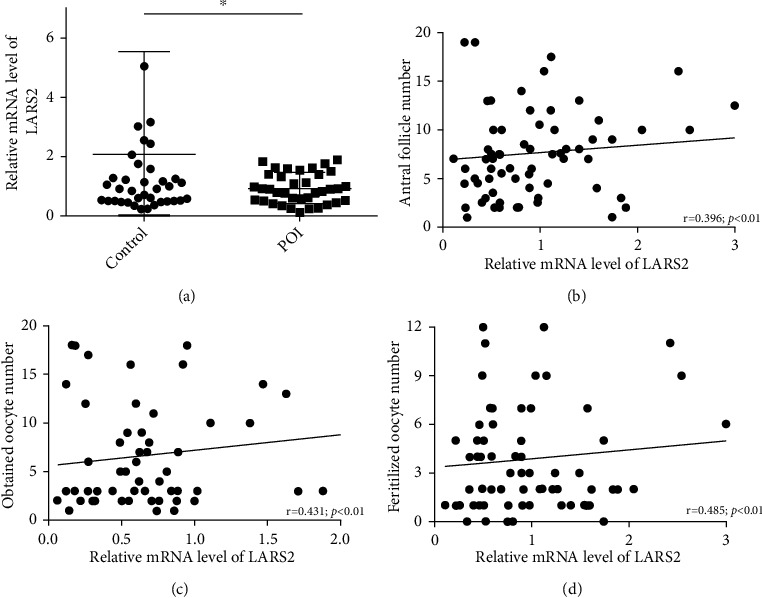
LARS2 levels are lower in POI patients than those in the control group. (a) The different levels of LARS2 between POI patients and control groups. (b–d) The correlation analysis of LARS2 levels with IVF outcomes, including antral follicle, obtained oocyte, and fertilized oocyte number. Person analysis for multiple comparisons of means. Meanwhile, for experiments involving only two groups, the data were analyzed by the two-sample *t*-test assuming unequal variances. ∗*P* < 0.05.

To explore the expression difference of LARS2 in granulosa cells, a total of 39 control and 37 POI patients were recruited. The characteristics of enrolled patients were shown in Table 1. All of the women were undergoing an IVF cycle in the hospital, and granulosa cells were collected after the oocyte was retrieved. The ovarian function of participants in the control group was normal, and women were undergoing an IVF cycle due to male factors or tubal obstruction. The POI patients exhibited typical endocrine profiles with elevated bFSH levels, and less AFC and oocytes were retrieved (Table 1). The expression changes of LARS2 in granulosa cell were determined using RT-qPCR. Our results showed that the expression levels of LARS2 were lower in the POI group compared to that in the control group (*P* < 0.05; Figure 1(a)). Meanwhile, in the correlation between LARS2 expression levels and the number of antral follicle, the number of obtained oocyte and fertilized oocyte was also analyzed in all 76 samples. The results showed that the expression levels of LARS2 were positively correlated with the antral follicle, obtained oocyte, and fertilized oocyte number, respectively (*P* < 0.01; Figures 1(b)–1(d)), indicating the essential role of LARS2 in follicle development.

### 3.2. LARS2 Promotes Cell Proliferation and Inhibits Apoptosis in Granulosa Cells

**Figure 2:**
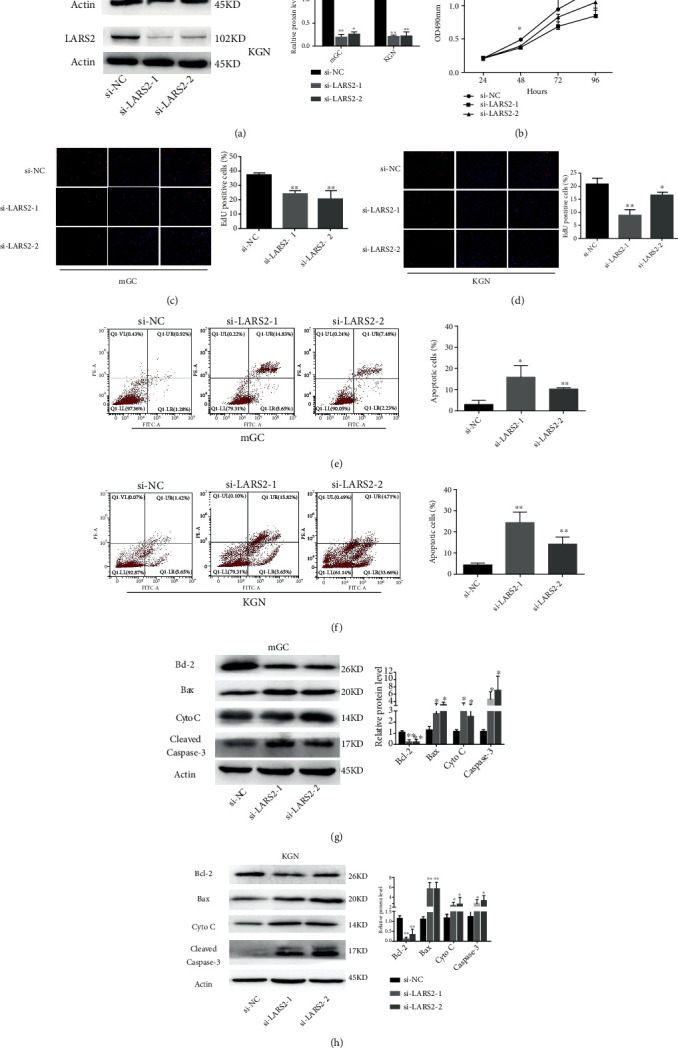
Biological effects of LARS2 on cell proliferation and apoptosis of KGN and mGC cells. LARS2 knockdown was performed by siRNAs interfering. (a) Level of LARS2 in KGN and mGC cells after siRNAs silencing were detected by western blot. (b) CCK8 assay was performed to detect cell viability of KGN. (c, d) Edu assay showed cell potential of DNA synthesis. l. (e, f) The apoptosis rate of KGN and mGC cells after transfection by si-LARS2. (g, h) Apoptosis-related proteins were detected by western blot after knockdown of LARS2. The data are presented as mean ± SD from three independent experiments. ∗*P* < 0.05.

It has been reported that LARS2 is involved in the regulation of cell proliferation and apoptosis in cancer cell [8]. To determine whether LARS2 affected granulosa cells physiology, the short interference RNA (siRNA) approach was utilized to knock down endogenous LARS2 expression. The knockdown efficiency was shown in Figure 2(a). The expression levels of LARS2 were significantly decreased in KGN cells and mouse granulosa cell (mGC) after the transfection of LARS2 siRNA compared to the negative control (*P* < 0.01; Figure 2(a)). CCK8 results showed that the cell viability of KGN was inhibited in LARS2 knockdown KGN cells (*P* < 0.05; Figure 2(b)). Meanwhile, Edu assay demonstrated that the DNA synthesis was decreased (*P* < 0.05; Figures 2(c) and 2(d)). Flow cytometry analysis suggested that silencing of LARS2 increased the percentage of apoptotic cells in both mGC and KGN cells (*P* < 0.05; Figures 2(e) and 2(f)). Moreover, western blot results showed that the protein levels of Bax, Cleaved Caspase-3, and cytochrome C were increased by knockdown of LARS2, whereas Bcl-2 protein levels were decreased (*P* < 0.05; Figures 2(g) and 2(h)). Concomitantly, administration of LARS2 siRNA could increase the level of cytosolic cytochrome C (*P* < 0.05; Figure S1c). Our results indicated that LARS2 inhibition affected granulosa cell proliferation and apoptosis.

### 3.3. LARS2 Knockdown Impairs Granulosa Cell Mitochondrial Function

**Figure 3:**
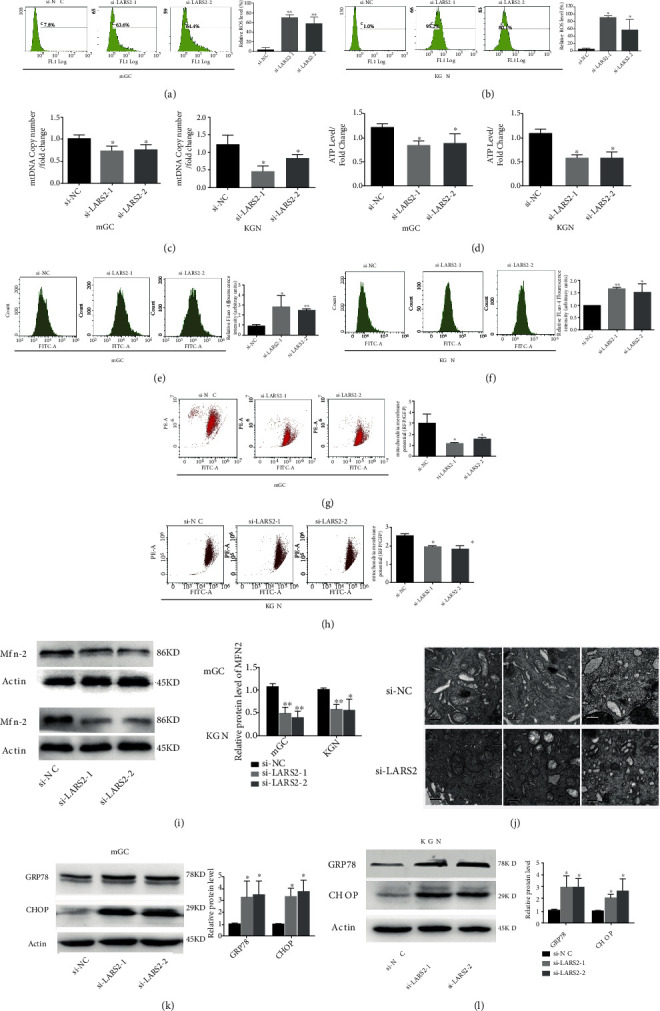
LARS2 effects mitochondrial function and dynamics in KGN and mGC cells. (a, b) Fluorescence intensity of DCFH-DA was used to measure ROS levels after transfection with si-LARS2. (c) mtDNA copy number was determined by qRT-PCR in KGN and mGC cells. (d) ATP levels were measured after treatment by si-LARS2. (e, f) Fluorescence intensity of Flou-4 was used to measure intracellular Ca^2+^ level after transfection with si-LARS2. (g, h) MMP was determined by using the fluorescent JC-1 with or without knocking down LARS2. (i) Mfn-2 levels were analyzed by western blot. KGN and mGC cells with or without si-LARS2. (j) Representative electron microscopic photographs of LARS2 knockdown cells. (k, l) Western blot detection of GRP78 and CHOP proteins expression in KGN and mGC cells transfected with si-LARS2 or si-NC, respectively. The data are presented as mean ± SD from three independent experiments. ∗*P* < 0.05.

In order to explore the effect of LARS2 on oxidative stress injury, both mGC and KGN cells were transfected with LARS2 siRNAs, and DCFH-DA staining was used to evaluate the production of ROS. As shown in Figures 3(a) and 3(b), LARS2 knockdown could significantly increase the production of ROS in mGC and KGN cells (*P* < 0.05). We investigated the mitochondrial ROS level by using Mito-SOX. Consistent with previous experimental results, LARS2 knockdown could increase the levels of mitochondrial ROS in KGN and mGC (*P* < 0.05; Figure S1a and S1b).

Meanwhile, the effect of LARS2 knockdown on granulosa cell mtDNA copy number and ATP levels were also determined. Our results showed that silencing of LARS2 had lower mtDNA copy number and ATP production in mGC and KGN cells compared with control, indicating that inhibiting LARS2 expression could impair mitochondrial activity via increasing oxidative stress in granulosa cell (*P* < 0.05; Figures 3(c) and 3(d)). It has been reported that oxidative stress can increase intracellular Ca^2+^ levels [12]. Accordingly, granulosa cells were stained with Fluo-4 AM, and Ca^2+^ levels were measured by flow cytometry. The results showed that LARS2 knockdown increased Ca^2+^ concentration in both mGC and KGN cells (*P* < 0.05; Figures 3(e) and 3(f)). As we know, excessive Ca^2+^ influx into cell mitochondria impairs mitochondrial function, as represented by a decrease in mitochondrial membrane potential (MMP) [13]. Hence, the changes in cellular MMP were detected, and the decreased MMP in granulosa cells was demonstrated when the levels of LARS2 expression were suppressed in the present study (*P* < 0.05; Figures 3(g) and 3(h)). Furthermore, the expression levels of mitochondria fusion protein were detected by western blot. As shown in Figure 3(i), downregulation of LARS2 could inhibit the expression of mitochondrial fusion-related gene mitofusin-2 (Mfn-2). In addition, transmission electron microscopy (TEM) technology was utilized to determine the morphology of mitochondria in KGN cells. Our results found that most of the mitochondrion was swollen, losing their cristae and containing large vacuoles (Figure 3(j)). Meanwhile, the endoplasmic reticulum (ER) showed an expansion morphology, characterized by multilamellar structures (Figure 3(j)). Collectively, these data suggested that LARS2 regulated the mitochondrial dynamics and function in granulosa cell.

### 3.4. LARS2 Mediates Endoplasmic Reticulum Stress in Granulosa Cell

As shown above, we demonstrated that ER structure was changed in LARS2 knockdown KGN cells. Meanwhile, the modulatory role of LARS2 on cell apoptosis was also found. Accordingly, we hypothesized that LARS2 may be involved in the regulation of apoptosis by activating ER stress signaling pathway. Our western blot results showed that LARS2 knockdown inhibited ER stress-related protein expression, including GRP78 and CHOP (*P* < 0.05; Figures 3(k) and 3(l)). These results indicated that LARS2 was involved in ER stress-induced apoptosis in granulosa cell.

### 3.5. NAC Attenuates LARS2 Knockdown-Induced Apoptosis and ER Stress in Granulosa Cell

**Figure 4:**
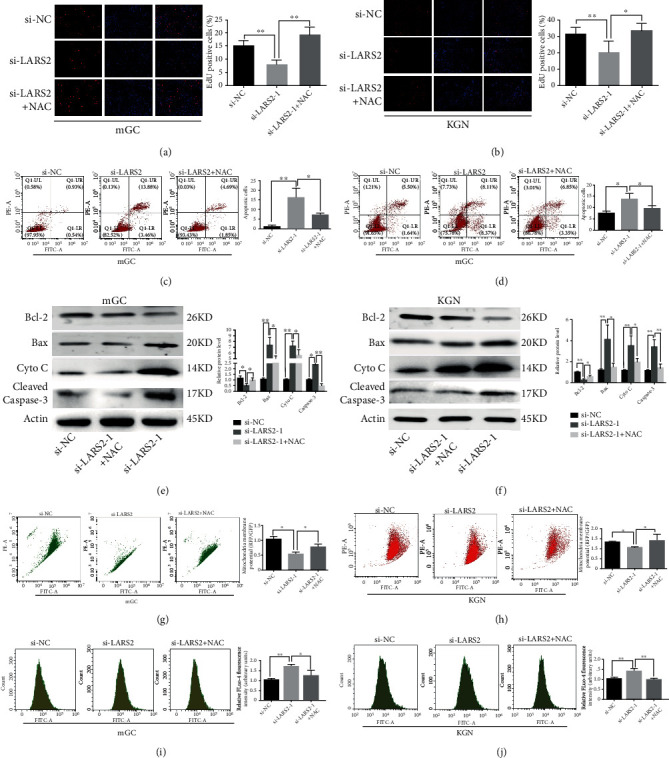
Effects of NAC treatment on LARS2-effected proliferation, apoptosis, and mitochondrial function in KGN and mGC cells. (a, b) Edu assay was performed to detect cell viability of KGN and mGC cells. Cells transfected with si-LARS2, with or without NAC(10 *μ*mol/L). (c, d) Cell apoptosis analysis carried out on KGN and mGC cells transfected with si-LARS2, with or without NAC. (e, f) Protein levels of apoptosis-related proteins in KGN and mGC cells transfected with si-LARS2 followed by NAC, as determined by western blot. (g, h) Flow cytometry with JC-1 staining were carried out on KGN and mGC cells, which had been treated in specific ways. Cells were transfected with si-LARS2 or si-NC, in the presence or absence of NAC. (i, j) ROS levels in KGN and mGC cells transfected with si-NC or si-LARS2 followed by NAC, as determined by flow cytometry. The data are presented as mean ± SD from three independent experiments. ∗*P* < 0.05.

**Figure 5:**
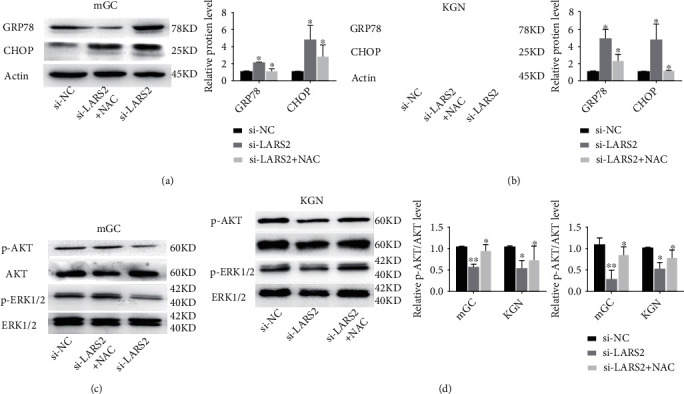
Effects of NAC treatment on LARS2 inhibited ERs and AKT/ERK cell signaling pathway. (a, b) Protein levels of GRP78, CHOP in KGN and mGC cells transfected with si-LARS2 followed by NAC, as determined by western blot. (c, d) Protein levels of AKT/ERK signaling pathway in KGN and mGC cells transfected with si-LARS2 followed by NAC, as determined by western blot. The data are presented as mean ± SD from three independent experiments. ∗*P* < 0.05.

More evidences indicated that excessive accumulation of ROS induces ER stress and mitochondrial dysfunction [14, 15]. N-acetyl-L-cysteine (NAC) is known as a commonly used active oxygen scavenger to decrease the cellular ROS level. To determine whether ROS mediates si-LARS2-induced cell death in granulosa cell, Edu and Annexin-FITC/PI staining analysis were used. The staining results found that the downexpression of LARS2 regulated cell proliferation and apoptosis in KGN and mGC cells; NAC treatment was found to promote these effects (*P* < 0.05; Figures 4(a)–4(d)). Moreover, western blot showed LARS2 knockdown-induced increase of apoptosis-related protein expression and decrease of antiapoptotic protein were partially reversed by NAC (*P* < 0.05; Figures 4(e) and 4(f)). Meanwhile, pretreatment NAC could reverse the blocking releasing of cytochrome c to the cytoplasm (*P* < 0.05; Figure S1d). We also demonstrated that NAC partly rescued the effect of LARS2 knockdown on MMP and Ca^2+^ influx in granulosa cells (*P* < 0.05; Figures 4(g)–4(j)). Additionally, the upregulation of GRP78, CHOP was also partially attenuated with NAC treatments in LARS2 siRNA-transfected cells (*P* < 0.05; Figures 5(a) and 5(b)). On all accounts, these results implied that the accumulation of ROS was involved in the ER-mediated antiproliferation and proapoptosis effects of LARS2 knockdown in granulosa cell.

### 3.6. LARS2 Knockdown Inhibits AKT and ERK Signing Pathways in Granulosa Cell

AKT and ERK1/2 are the two crucial downstream signaling pathways responding to gonadotropin stimulation. The disruption of AKT and ERK1/2 will cause female infertility [16, 17]. After the gonadotropin surge, these two signaling pathways will be activated in a phosphorylated manner. To explore the effect of LARS2 on AKT and ERK1/2 phosphorylation, the expression of LARS2 were inhibited through transfecting specific siRNA in both mGC and KGN cells. The results showed that LARS2 knockdown significantly decreased the phosphorylation levels of AKT and ERK1/2 (*P* < 0.05; Figures 5(c) and 5(d)). Importantly, the NAC supplement partially reversed LARS2 knockdown-induced downregulation of AKT and ERK1/2 phosphorylation (*P* < 0.05; Figures 5(c) and 5(d)).

### 3.7. Transcriptome Analysis in LARS2 Knockdown KGN Cell

**Figure 6:**
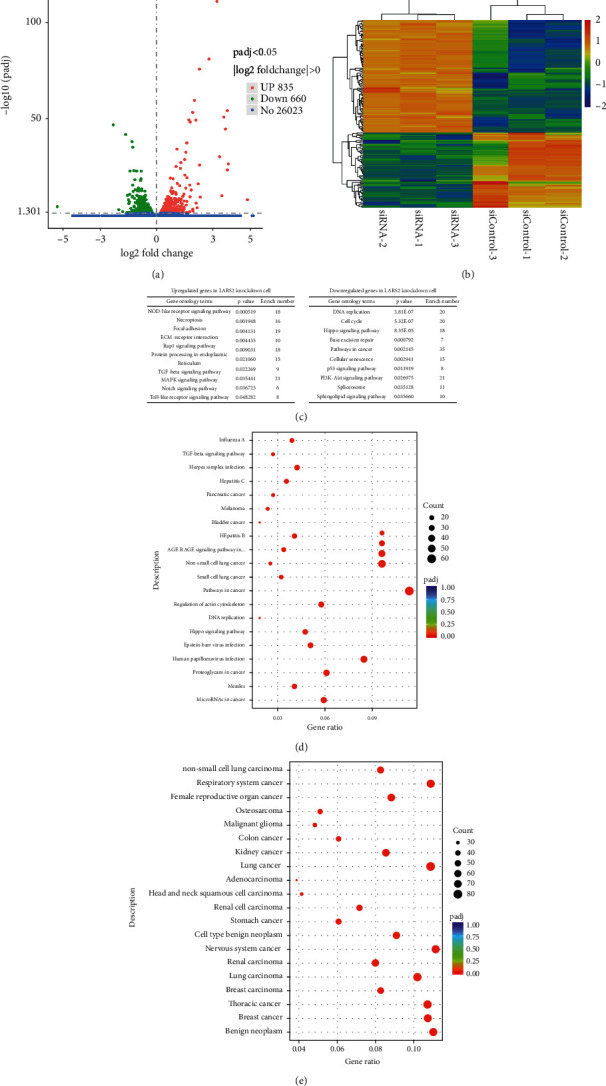
Gene expression is altered in KGN cells after LARS2 knockdown. (a) Volcano plots for RNA-seq comparing si-LARS2 and si-NC KGN cell line. Red spot represents –log2 (*P*-value) ≥0; blue spot represents the -log2 (*P*-value) <0. (b) Heatmap for differentially expressed genes in KGN after transfected with si-LARS2 or si-NC. The color spectrum ranging from red to blue indicates normalized levels of gene expression from high to low. The inclusion criteria of were |–log10 | < 1. (c) GO enrichment of differentially expressed genes between si-LARS2 and control KGN cells. (d) KEGG pathway analysis of differentially expressed genes in si-LARS2 compared to control. (e) The enrichment analysis of human disease-related genes using the Disease Ontology method. The data are presented as mean ± SD from three independent experiments. ∗*P* < 0.05.

To further identify the potential key factor involved in the molecular function of LARS2, RNA sequencing (RNA-seq) technology was used to explore the differential expression genes (DEGs) in LARS2 knockdown KGN cells. Totally, there are 1495 DEGs, including 835 upregulated genes and 660 downregulated genes (Figures 6(a) and 6(b)). Furthermore, the transcriptional changes were discussed at overall level by GO (Gene Ontology) and KEGG classification analysis. GO analysis showed that these altered genes are enriched in the activation of MAPK activity, extrinsic apoptotic signaling pathway, etc. (Figure 6(c)). KEGG pathway analysis indicated that the changed genes were enriched for DNA replication, TGF-beta signaling pathway, and cellular senescence (Figure 6(d)). Meanwhile, the Disease Ontology (DO) analyses were also performed to explore the change of genes involved in human disease (Figure 6(e)).

### 3.8. E2F1 Regulates Mfn-2 Expression in Granulosa Cell

**Figure 7:**
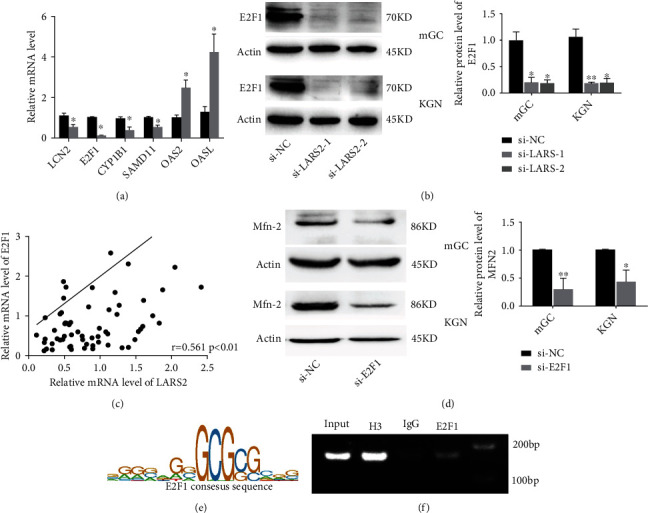
E2F1 regulate Mfn-2 transcription in ovarian granulosa cells. (a) The relative mRNA expressions of genes after treatment by si-LARS2 in KGN cell line. (b) The protein level of E2F1 in KGN and mGC cells after transfected with si-LARS2, as determined by western blot. (c) Correlations between the LARS2 levels and the mRNA levels of E2F1 in ovarian granulosa cells from patients. (d) Level of Mfn-2 in KGN and mGC cells after treatment by si-E2F1 were detected by western blot. (e) The sequence logo of a potential E2F1 binding site in JASPAR. (f) ChIP assay was performed using E2F1 antibody to determine the E2F1 binding to the Mfn-2 gene promoter. The data are presented as mean ± SD from three independent experiments. ∗*P* < 0.05.

Based on the RNA-seq result, several DEGs were selected to determine the expression level changes in LARS2 knockdown cells using RT-qPCR, including LCN2, E2F1, CYP1B1, SAMD11, OAS2, and OASL. As shown in Figure 7(a), the expression levels of LCN2, E2F1, CYP1B1, and SAMD11 were decreased in LARS2 knockdown cells, whereas OAS2 and OASL expression were increased, which were consisted with the RNA-seq results (*P* < 0.05). Meanwhile, the protein changes of E2F1 were further confirmed by western blot in both mGC and KGN cells. Similar to RT-qPCR results, knockdown of LARS2 significantly decreased E2F1 protein expression levels (*P* < 0.05; Figure 7(b)). Moreover, we also found that the mRNA levels of E2F1 were positively correlated with the LARS2 mRNA levels in granulosa cells from all 76 patients (*R* = 0.561; *P* < 0.01; Figure 7(c)). E2F1 is a member of the E2F transcription factor family, which is involved in cell cycle regulation through the cyclin-dependent interaction with pocket proteins [18]. Furthermore, it has been proved that E2F1 could promote the expression of Mfn-2 in HeLa cell [19]. To examine whether E2F1 regulated Mfn-2 in granulosa cell, the protein levels of Mfn-2 were detected after the transfection of E2F1 siRNA in both mGC and KGN cells. Consistent with the previous reports, western blot results showed that E2F1 knockdown decreased the expression levels of Mfn-2 in granulosa cells (*P* < 0.05; Figure 7(d)). Additionally, online prediction results showed that there were putative E2F1-binding sites in the Mfn-2 gene promoter (Figure 7(e)). The binding capacity of E2F1 on the Mfn-2 promoter was determined by ChIP assay. The results indicated that E2F1 could bind with the Mfn-2 promoter (*P* < 0.01; Figure 7(f)), indicating the transcriptional regulation effect of E2F1 on Mfn-2 expression in granulosa cell.

### 3.9. GH Alleviates Doxorubicin-Induced POI-like Mouse Phenotype

**Figure 8:**
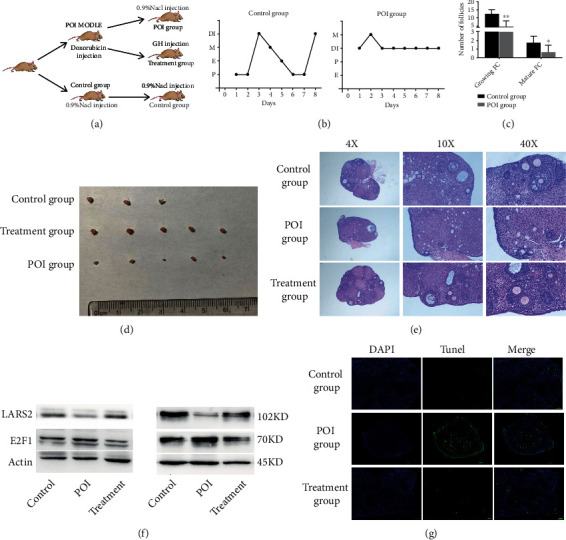
Effect of GH on POI mouse model. (a) The diagram of animal experiment procedure. (b) The stage analysis of mouse estrus cycle in different groups. Proestrus stage was abbreviated as “P”; estrus stage was abbreviated as “E”; metestrus stage was abbreviated as “M”; diestrous stage was abbreviated as “DI”. (c) Follicle counts in POI and control group mice ovaries. The number of follicle subtypes per ovary was quantified as described in the Materials and Methods section. (d) The ovaries for different group mice were shown. (e) H&E staining of representative ovaries. (f) The expression changes of LARS2 and E2F1 in different groups. (g) Granulosa cell apoptosis in ovarian sections from each group was measured by fluorescent TUNEL staining. Green fluorescences indicate TUNEL-positive apoptotic cells TUNEL staining of representative ovaries. The data are presented as mean ± SD from three independent experiments. ∗*P* < 0.05.

To explore the involvement of LARS2 in the occurrence of POI in vivo, the POI mouse model was constructed. Three-week-old C57 mice were continuously given an intraperitoneal injection using doxorubicin. The animal experiment procedure was shown in Figure 8(a). After 3-week injection, the estrus cycles were arrested at the diestrous (DI) stage in the POI group, whereas the control group mice had normal estrus cycles (Figure 8(b)). Ovarian section analysis revealed that POI group mice had a smaller number of growing follicles (primordial, primary, and secondary) and mature follicles (*P* < 0.05; Figure 8(c)). These results proved that POI mice were successfully established. Clinically, growth hormone (GH) is used to improve oocyte quality. GH is a common antioxidant drug that can reduce ROS levels and improve mitochondrial function in vivo. Considering the role of GH in decreasing ROS levels, POI mice were treated with GH to evaluate whether the POI symptoms could be reversed by GH. Our results showed that the ovary size in the POI group was smaller than the control mice (Figure 8(d)). Intriguingly, the ovary size in the GH treatment group was bigger than those in POI group mice (Figure 8(d)). HE staining results showed that GH treatment group mice had increased the number of growing and mature follicles compared to POI mice even though abnormal follicles were still higher compared with these in control group (*P* < 0.05; Figure 8(e)). Furthermore, we detected the expression level changes of the target protein in the ovary. The expression levels of LARS2 in POI mice were decreased, whereas E2F1 expression were increased (*P* < 0.05; Figure 8(f)). TUNEL assay showed that increased percentage of total apoptotic cells were detected in the POI group and markedly ameliorated by GH (*P* < 0.05; Figure 8(g)). Collectively, animal experiment results further demonstrated that LARS2 was involved in the occurrence of POI. Importantly, GH therapy could significantly improve the POI phenotype.

## 4. Discussion

Excessive follicular atresia is closely correlated with the occurrence and development of POI. Several studies indicate that the abnormal physiology of granulosa cell drives the atresia of follicle [20, 21]. Accelerated granulosa cells apoptosis may result in premature cessation of ovarian function [22]. In the present study, we demonstrated the expression levels of LARS2 were decreased in POI patients compared to the control group. Furthermore, our study found that the downregulation of LARS2 inhibited cell proliferation and promoted cell apoptosis in KGN cells and mGC. Our results indicated that aberrant expression of LARS2 contributed to the occurrence of POI via impair granulosa function, in which induced the excessive granulosa cell apoptosis.

Previous studies have shown that the mitochondrial apoptotic pathway is one of the principal pathways leading to cell apoptosis [23, 24]. Dysfunction of mitochondria induces intrinsic apoptotic cell death program, including chromosome fragmentation and caspase activation. The initiator caspase for the mitochondrial-mediated apoptotic pathway is caspase-9 [25, 26]. Then, mitochondrial-released cytochrome C induces the formation of apoptosome. Apoptosome contains several other cytochrome C homologs, procaspase-9, and the adaptor protein Apaf-1 to support the activation of caspase-9, resulting in the subsequent degradation of cellular death substrates [27, 28]. In the present study, the Bax/Bcl-2 ratio was increased by LARS2 silencing. In addition, activation of caspase-3 and cytoplasmic cytochrome C were also detected. These results proved that LARS2 effected mitochondria-mediated apoptosis in KGN cells and mGCs.

Mitochondria are known as an important intracellular organelle for producing ROS. ROS has dual functions in cellular physiology depending on the cellular concentration. Excessive level of ROS causes a variety of biological dysfunction, including oxidative stress, accumulation of mtDNA mutations, and disruption of Ca^2+^ homeostasis in the cytosol, even leading to ER stress [29–32]. ROS is a double-edged sword in ER stress. ROS could be responding to ER stress to unfolded protein response (UPR), which helps to alleviate the protein burden in ER [31]. However, excessive activation of URP process induces several proapoptotic proteins expression, like CHOP. Then CHOP induces the secondary rise of ROS level in the cell. This secondary increase in the level of ROS and upregulated CHOP expression will induce cell death via activating apoptosis pathway [30]. In our present study, the downregulation of LARS2 induced ER stress by elevating the expression levels of GRP78, CHOP protein in granulosa cells. ROS is also known as a key factor in cellular senescence [33]. The cumulative effect of ROS causes aging and plays a pivotal role in various degenerative diseases [34]. Therefore, ROS is an effective indicator of cell apoptosis [35]. In fact, the high level of ROS in POI patient granulosa cell has been reported [36, 37]. In the current study, we explored the changes of ROS level in LARS2 knockdown granulosa cells, and higher ROS levels were demonstrated. Importantly, active oxygen scavenger NAC could partially reverse LARS2 knockdown-induced cell apoptosis, indicating that ROS plays a key role in LARS2-regulating mitochondrial apoptosis. Meanwhile, the underlying mechanism of LARS2-effected mitochondrial dysfunction was also explored in the present study. Our results showed that LARS2 knockdown decreased MMP, mtDNA, and ATP production in granulosa cells. These changes might be probably linking to mitochondrial dysfunction in LARS2 knockdown granulosa cells. Previous studies have proved that mtDNA copy number is closely related with POI [38, 39]. The normal mitochondria function guarantees the follicle development and affects fertilization outcomes [5, 40, 41]. In view of the above, low expression of LARS2 results in a compromised mitochondrial stress response, increased ROS level, and accelerated granulosa cell death, which is contributed to the occurrence of POI.

It has been reported that mitochondria are dynamic organelles that continuously move, fuse, and divide [42]. A dynamic balance between fusion and fission is essential for the maintenance of mitochondrial function and cellular homeostasis [43, 44]. In particular, mitochondrial dynamics is also a crucial process not only for mitochondrial morphology but also in the control of mitochondrial function responding to apoptotic stimuli [45–47]. Malfunctioning of mitochondrial fusion and fission causes various human pathological changes, including reduced fertility [48, 49]. Anchoring on the outer membrane of mitochondrial, Mfn-2 plays an important role in mediating mitochondrial fusion [50]. Loss of function of Mfn-2 impairs the cellular mitochondrial dynamic balance, which leads to the inhibition of cell proliferation by disrupting MMP and decreasing respiration and ATP production [51, 52]. Besides the role in mitochondrial fusion, Mfn-2 has been described to be involved in the regulation of cell autophagy, mitochondrial antiviral signaling, and unfolded protein response [53]. A previous study has suggested that lacking Mfn-2 increased apoptosis, resulting in compromised oocyte quality and accelerated follicular depletion in mouse [48]. Meanwhile, a clinical investigation has reported that the lower expression levels of Mfn-2 in human granulosa cells are correlated with the clinical outcome of ART by affecting mitochondrial function [54]. In the current study, the effect of downregulated LARS2 expression on mitochondrial dynamics in granulosa cell was also explored. Our results found that knockdown of LARS2 could disrupt mitochondrial dynamics by decreasing the expression of Mfn-2 in granulosa cells. In addition, we also demonstrated that E2F1 mediated the effect of LARS2 on Mfn-2 expression via targeting the promoter region of Mfn-2 to promote transcription.

**Figure 9:**
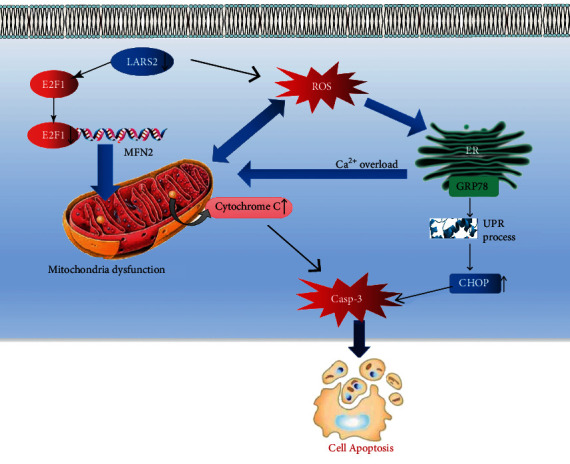
Schematic map concerning the functional role of LARS2 in POI. In the present study, we demonstrated that the downregulation of LARS2 induced granulosa cell apoptosis. Mechanically, our results showed that two different pathway were involved in LARS2-meidiated granulosa cell apoptosis. (I) Aberrant decrease of LARS expression impaired granulosa cell mitochondria function via inhibiting E2F1 mediated the mitochondrial motor protein MFN2 expression. Subsequently, the decreased MFN2 expression induced mitochondria dysfunction. The persistent mitochondria dysfunction promoted the release of cytochrome C. The accumulation of cytoplasmic cytochrome C activated mitochondrion-mediated cell apoptosis. (II) The dysregulation of LARS2 expression induced the increase of reactive oxygen species (ROS) level in granulosa cell. The upregulation of ROS level caused mitochondria dysfunction, which leads to the accumulation of cytoplasmic cytochrome C. On the other hand, the increased ROS level induced the occurrence of endoplasmic reticulum (ER) stress, which provoked ER stress unfolded protein response (UPR). The activation of UPR upregulated apoptotic protein CHOP expression and promoted cell apoptosis.

In summary, the present study demonstrates that the levels of LARS2 expression are decreased in POI patient granulosa cells. LARS2 knockdown inhibits cell proliferation and induces apoptosis in granulosa cell. The inhibition of LARS2 impairs granulosa cell mitochondrial function by increasing ROS levels and decreasing mtDNA copy number and ATP levels. Meanwhile, our study also finds that LARS2 knockdown could impair mitochondrial dynamics via inhibiting the expression of Mfn-2. Furthermore, LARS2 mediates ER stress in granulosa cell by regulating ER stress-related protein expression. Interestingly, the supplementary of NAC could partially rescue LARS2 knockdown-induced apoptosis in granulosa cell (Figure 9). Transcriptomic profiles reveal that the E2F1 is downregulated in LARS2 knockdown KGN cells. E2F1 mediates the effect of LARS2 on Mfn-2 expression via targeting the promoter region of Mfn-2 to promote transcription. Moreover, the phosphorylated levels of AKT and ERK1/2 (two important signaling pathways responding to gonadotropin) are decreased when LARS2 expression is inhibited. Additionally, our study demonstrates that GH treatment alleviates doxorubicin-induced POI-like mouse phenotype. Our results highlight the potential role of LARS2 in the occurrence of POI and provide the new insight of molecular mechanism in LARS2-mediated mitochondrial dysfunction and cell apoptosis, which may be a benefit to the clinical therapeutics of POI.

## Figures and Tables

**Table 1:** Clinical characteristics of patients with biochemical POI and controls.

Characteristics	Control (*n* = 39)	POI (*n* = 37)	*P* value
Baseline characteristics			
AGE	29.79 ± 2.65	36.81 ± 5.53	<0.001∗∗
Basal FSH (mIU/mL)	6.39 ± 1.13	8.84 ± 3.39	<0.001∗∗
Basal LH (mIU/mL)	4.72 ± 2.12	4.31 ± 1.79	0.375
Basal estradiol (pg/mL)	127.26 ± 62.01	145.26 ± 93.71	0.335
AFC	10.83 ± 4.91	5.19 ± 3.58	<0.001∗∗
AMH(ng/mL)	3.07 ± 0.79	0.58 ± 0.25	<0.001∗∗
IVF treatment cycle parameters			
Number of follicles on day of HCG (≥14 mm)	10.38 ± 3.43	3.7 ± 1.37	<0.001∗∗
Obtained oocyte number	13.28 ± 4.62	3.22 ± 1.86	<0.001∗∗
Fertilized oocyte number	6.44 ± 3.94	1.75 ± 1.44	<0.001∗∗

Data are presented as mean ± SD values. AFC: basal antral follicle count; FSH: follicle-stimulating hormone; LH: luteinizing hormone. AMH: anti-Müllerian hormone. ∗*P* < 0.05 versus control; ∗∗*P* < 0.01 versus control.

**Table 2:** Primers of real-time PCR (RT-PCR), chromatin immunoprecipitation (ChIP) assay.

Gene	Primer sequence (forward)	Primer sequence (reverse)
LARS2	GTGCTTTCCATGTTCCCTTATC	GTGCTTTCCATGTTCCCTTATC
LARS2(*mus*)	GCAGACAAGGAAGGATGTGGAGAAG	GCTTGCCAGAAGGGTATGGGAAC
GAPDH	CATGTTCGTCATGGGTGTGAACCA	AGTGATGGCATGGACTGTGGTCAT
GAPDH(*mus*)	AGGTCGGTGTGAACGGATTTG	GGGGTCGTTGATGGCAACA
mtDNA	CACCCAAGAACAGGGTTTGT	TGGCCATGGGTATGTTGTTA
mtDNA(*mus*)	TACCTCACCATCTCTTGCTA	CCACATAGACGAGTTGAT-TC
*β*2M	TGCTGTCTCCATGTTTGATGTATCT	TCTCTGCTCCCCACCTCTAAGT
*β*2M(*mus*)	GAACTGCTACGTAACACAGTTC	GTATGTATCAGTCTCAGTGGGG
E2F1	ATAGTGTCACCACCACCATCAT	GAAAGGCTGATGAACTCCTCAG
E2F1(*mus*)	CACTAAATCTGACCACCAAACG	CATTGGTGATGTCATAGATGCG
Primers used in ChIP		
E2F1-p1 (+96/+106)	TTAGCCAGGCATGGTGGT	ATCTTGGCTCACCGCAAC
E2F1-p2 (+536/+543)	CAGGACAGCTCCCTTTGG	TTCTGTGGGATGGTGCTG
E2F1-p3 (+976/+986)	TGGGTAACAAAGTGAAATGC	GCTCAAGTAATCCTCCCAC
E2F1-p4 (-123/+41)	GTGGGAGTCCGAGCCTCT	GAGCTGGTGGACCCTGAG
E2F1-p5 (-371/-200)	CCGATGAGTCACTTCACCCTA	CTGTCAAGGGGCGAAAAAC

## Data Availability

The data are available on request from the corresponding author, Long Bai, through email: bailong0375@zju.edu.cn or Yimin Zhu through email: zhuyim@zju.edu.cn

## Supplementary Materials

Supplementary Materials Figure S1: LARS2 effects mitochondrial function and cytoplasmic CytoC levels. (a, b) Fluorescence intensity of Mito-SOX was used to measure mitochondrial ROS levels after transfection with si-LARS2. (c) Cytoplasmic CytoC was detected by western blot after knockdown of LARS2. (d) Protein levels of cytoplasmic CytoC in KGN and mGC cells transfected with si-LARS2 followed by NAC, as determined by western blot. The data are presented as mean ± SD from three independent experiments. ∗*P* < 0.05.
