# Supplementary material for: LARS2 Regulates Apoptosis via ROS-Mediated Mitochondrial Dysfunction and Endoplasmic Reticulum Stress in Ovarian Granulosa Cells
Source: Oxid Med Cell Longev. 2022 May 9;2022:5501346. doi: 10.1155/2022/5501346 (PMC9110257; doi:10.1155/2022/5501346)
Supplement: Supplementary Materials — Figure S1: LARS2 effects mitochondrial function and cytoplasmic CytoC levels. (a, b) Fluorescence intensity of Mito-SOX was used to measure mitochondrial ROS levels after transfection with si-LARS2. (c) Cytoplasmic CytoC was detected by western blot after knockdown of LARS2. (d) Protein levels of cytoplasmic CytoC in KGN and mGC cells transfected with si-LARS2 followed by NAC, as determined by western blot. The data are presented as mean ± SD from three independent experiments. ∗P < 0.05. [file 5501346.f1.zip › Supplementary Information.docx]

**Supplementary Information**

**FigureS1 LARS2 effects mitochondrial function and cytoplasmic Cyto C levels**

(a,b) Fluorescence intensity of Mito-SOX was used to measure mitochondrial ROS levels after transefection with si-LARS2. (c) Cytoplasmic CytoC was detected by western blot after knockdown of LARS2. (d) Protein levels of Cytoplasmic CytoC in KGN and mGC cells transfected with si-LARS2 followed by NAC, as determined by Western blot. The data are presented as mean±SD from three independent experiments. **P* < 0.05.
